# Women with a positive high-risk human papillomavirus (HPV) test remain at increased risk of HPV infection and cervical precancer ≥15 years later

**DOI:** 10.1016/j.tvr.2022.200240

**Published:** 2022-05-28

**Authors:** Federica Inturrisi, Johannes A. Bogaards, Albert G. Siebers, Chris J.L.M. Meijer, Daniëlle A.M. Heideman, Johannes Berkhof

**Affiliations:** a Amsterdam UMC Location Vrije Universiteit Amsterdam, Epidemiology and Data Science, De Boelelaan, 1117, Amsterdam, the Netherlands; b Amsterdam Public Health, Methodology, Amsterdam, the Netherlands; c PALGA, 3991, SZ Houten, the Netherlands; d Amsterdam UMC Location Vrije Universiteit Amsterdam, Pathology, De Boelelaan, 1117, Amsterdam, the Netherlands; e Cancer Center Amsterdam, Imaging and Biomarkers, Amsterdam, the Netherlands

**Keywords:** Human papillomavirus (HPV), Genotype, Cervical intraepithelial neoplasia (CIN), Cervical screening, Screening interval, ≥ASC-US, atypical squamous cells of undetermined significance or worse, CI, confidence interval, ≥CIN2, cervical intraepithelial neoplasia grade 2 or worse, ≥CIN3, cervical intraepithelial neoplasia grade 3 or worse, EIA, enzyme immunoassay, hrHPV, high-risk human papillomavirus, OR, odds ratio, PALGA, nationwide network and registry of histo- and cytopathology in the Netherlands, Dutch for “Pathologisch-Anatomisch Landelijk Geautomatiseerd Archief”, POBASCAM, Population-based Screening Study Amsterdam

## Abstract

Little is known about the long-term association between high-risk human papillomavirus (hrHPV) test results in women participating in a hrHPV-based cervical cancer screening program. To address this question, we collected data of 2217 women who participated in the POBASCAM hrHPV-based screening trial (enrolment 1999/2002) and also attended the Dutch hrHPV-based screening program between January 2017 and March 2018. Among 143 women who tested hrHPV-positive in 1999/2002, 45 (31.5%) had ≥ CIN2 or hysterectomy before 2017 and 17 (11.9%) tested hrHPV-positive at the 2017/2018 screen. In comparison, among 2074 women who tested hrHPV-negative in 1999/2002, 10 (0.5%) had ≥ CIN2 or hysterectomy before 2017 and 119 (5.7%) tested hrHPV-positive at the 2017/2018 screen. It follows that in the group of women who were not treated for ≥ CIN2 or had a hysterectomy in between the two screens 15 years apart (N = 2162), women who were hrHPV-positive in 1999/2002 had a higher risk of being hrHPV-positive in 2017/2018 than those who were hrHPV-negative in 1999/2002 (OR 3.4, 95% CI 1.8–6.1). A similar association was found at the genotype level for genotype-concordant results (5.1, 1.0–11.3) and for genotype non-concordant results (3.7, 1.6–6.7). Women who were hrHPV-positive in 2017/2018 had a higher risk of CIN3 after a hrHPV-positive result in 1999/2002 than after a hrHPV-negative result (5.8, 1.0–27.8). In conclusion, a positive hrHPV result in screening gives a long-term increased risk of a hrHPV-positive result, also for different genotypes, and a long-term increased risk of CIN3. This supports the concept of risk-stratification in hrHPV-based cervical cancer screening where previous hrHPV results are included in screening recommendations.

## 1 Introduction

High-risk human papillomavirus (high-risk HPV, hrHPV) infections are responsible for virtually all cervical cancers [1]. Cervical hrHPV prevalence is highest around the age of sexual debut and decreases with age [2]. In most settings, a decrease in hrHPV DNA detection rates in middle age is followed by a second peak in the peri- or post-menopausal years [3].

Little is known about the association between long-term longitudinal hrHPV test results in cervical cancer screening populations. This type of information may contribute to our understanding of the natural history of hrHPV infections [4] and may be used to optimize hrHPV-based screening programs [5]. In current hrHPV-based screening programs, screening intervals of 10 (the Netherlands) and 7 years (Sweden) after a negative hrHPV test have already been implemented for women with age ≥40 and ≥ 50, respectively [6]. We expect that efficiency and effectiveness of programs can be further improved by stratifying women by previous hrHPV screening results [7].

In this study, we analyzed hrHPV and histology data from 2217 women who participated in the POBASCAM hrHPV-based screening trial with enrolment from 1999 to 2002 [8] and also attended the Dutch hrHPV-based screening program in 2017/2018. The aim was to assess the association between the hrHPV result and the risk of genotype-specific HPV and cervical intraepithelial neoplasia (CIN) grade 3 or worse (≥CIN3) 15–20 years later. HrHPV genotyping information was collected in all women included in this study at the two screens, in the enrolment screen of the POBASCAM trial in 1999/2002 and in the 2017/2018 screen. In our main analysis, we only included women who were not treated for CIN grade 2 or worse (≥CIN2) or had a hysterectomy in between the two screens.

## 2 Material and methods

### 2.1 Study design and data source

This is a cohort nested within the national hrHPV-based cervical cancer screening program of the Netherlands. The population consists of 2217 women who participated in the POBASCAM hrHPV-based screening trial and also attended the hrHPV-based screening program between January 2017 and March 2018 15–20 years later.

POBASCAM is a randomized trial embedded in the (cytology-based) screening program in the Netherlands [[8], [9], [10]]. Between January 1999 and September 2002, eligible consenting women (N = 44102) aged 29–61 were randomly assigned (1:1) to receive hrHPV and cytology co-testing (intervention group) or cytology-only with blinded hrHPV testing (control group). The new hrHPV-based screening program was implemented in the Netherlands in January 2017. Here, hrHPV-positive women are managed by reflex cytology and repeat cytology after 6 months. Women with abnormal cytology (≥ASC-US) are referred for colposcopic evaluation. Women from the POBASCAM cohort who turned 45, 50, 55 or 60 in 2017 were invited for their fourth program visit after enrolment.

All screening test results in the Netherlands are stored in the nationwide network and registry of histo- and cytopathology (PALGA, Houten, NL). We identified cervical screening samples (for hrHPV testing) of 2217 women who participated in both the POBASCAM trial and the national screening program between January 2017 and March 2018. POBASCAM enrolment test results were retrieved from women assigned to both study groups (1065 from the intervention and 1152 from the control group). For hrHPV-positive women at the 2017/2018 screen (N = 143), cervical samples were collected and genotyped and histological follow-up was collected up to January 2021.

This study was approved by the scientific committee of PALGA. The POBASCAM trial (Trial registration ID: NTR218) was approved by the Medical Ethics Committee of the VU University Medical Centre (Amsterdam, NL; no 96/103) and the Ministry of Public Health (The Hague, NL; VWS no 328650). All women in the POBASCAM study gave written informed consent.

### 2.2 Laboratory procedures

HrHPV DNA testing in the POBASCAM study was done using GP5+/6+ PCR-enzyme immunoassay (EIA) [11] which uses an oligonucleotide probe cocktail that detects any of 14 hrHPV genotypes (i.e., genotypes 16/18/31/33/35/39/45/51/52/56/58/59/66/68). HrHPV DNA testing in the 2017/2018 screen was done by the Cobas HPV Test (Cobas 4800 System, Roche Molecular systems, Branchburg, US) which tests for the same hrHPV genotypes as the GP5+/6+ PCR-EIA test. HrHPV-positive samples at the 2017/2018 screen (N = 143) were retested by GP5+/6+ PCR-EIA. Subsequent genotyping of EIA-positive samples was conducted using a reverse line blot assay (POBASCAM study) [11] or microsphere bead‐based assay (Luminex; 2017/2018 screen) [12]. Samples that failed to show a positive signal in the typing assay were designated as “genotype-negative”.

Histology was examined routinely and classified as normal, CIN grade 1, 2, 3, or invasive cancer. Adenocarcinoma in situ was added to CIN3.

### 2.3 Data analysis

We studied associations between hrHPV screening results of the 1999/2002 and 2017/2018 screen for i. generic hrHPV test results, ii. HPV16 results, iii. hrHPV genotype-concordant results, and iv. hrHPV genotype non-concordant results. For iii. the genotype-concordant results, we constructed a two way frequency table for every genotype with variable “hrHPV genotype reported in 1999/2002 (yes/no)” by variable “hrHPV genotype reported in 2017/2018 (yes/no)” and subsequently pooled over genotypes using the Mantel-Haenszel odds ratio (OR) formula. For iv. the genotype non-concordant results, we selected women who were either hrHPV genotype-positive or hrHPV-negative and constructed a two way frequency table for every genotype with variable “hrHPV genotype reported in 1999/2002 (yes/no)” by variable “at least one hrHPV genotype other than 1999/2002 genotype reported in 2017/2018 (yes/no)” and again pooled over genotypes using the Mantel-Haenszel OR formula.

**Fig. 1:**
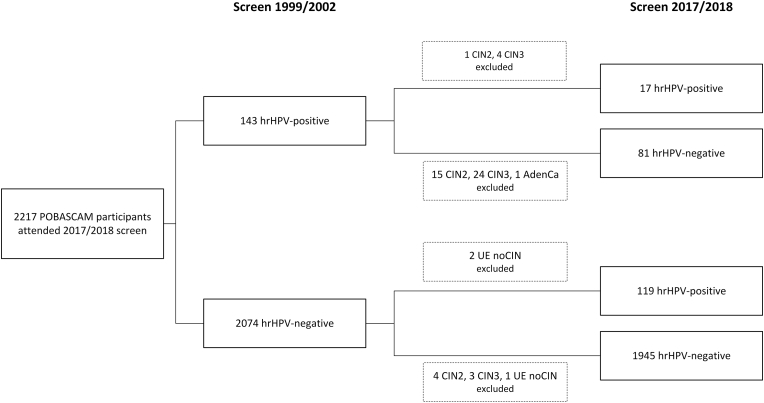
Flowchart of study sample, with hrHPV test results at the 1999/2002 screen and at the 2017/2018 screen. Women with ≥CIN2 or hysterectomy between the two screens were excluded from the main analysis. Abbreviations: AdenCa: adenocarcinoma in situ; CIN, cervical intraepithelial neoplasia; hrHPV, high-risk human papillomavirus; UE: uterus extirpation (hysterectomy).

Women with ≥CIN2 or hysterectomy diagnosed between the 1999/2002 screen and the 2017/2018 screen were excluded from the main analysis (N = 55). A flowchart of the study population included in the main analysis is presented in Fig. 1. We repeated the analyses for women aged <50 and ≥ 50 years at the 2017/2018 screen, and all women including those with ≥CIN2 or hysterectomy before 2017. Finally, we analyzed the subgroup of women with a positive hrHPV test in 2017/2018 with respect to ≥ CIN3 risk where we stratified according to hrHPV status in 1999/2002.

We calculated ORs with 95% confidence intervals (CI). For the pooled genotype level analyses, we calculated Mantel-Haenszel estimates with 95% confidence bootstrap intervals to account for genotype clustering within individuals. Differences in risks were evaluated by means of Fisher's exact test and p < 0.05 was considered statistically significant. Analyses were performed using STATA 14.1 and R software version 3.6.1.

## 3 Results

The mean age of the 2217 women was 37 years (range 29–45) at the 1999/2002 screen and 53 years (range 44–63) at the 2017/2018 screen. The mean time between the two screens was 15.7 years (range 14.7–19.4). Of the 2074 hrHPV-negative women at the POBASCAM enrolment screen in 1999/2002, 10 (0.5%) had ≥ CIN2 or hysterectomy before 2017 and of the remaining women, 1945 (93.8%) tested hrHPV-negative and 119 (5.7%) tested hrHPV-positive at the 2017/2018 screen (Fig. 1). Of the 143 hrHPV-positive women in 1999/2002, 45 (31.5%) had ≥ CIN2 or hysterectomy before 2017 and of the remaining women, 81 (56.6%) tested hrHPV-negative and 17 (11.9%) tested hrHPV-positive at the 2017/2018 screen. In the latter group, 4 had genotype(s) also detected at the 1999/2002 screen, 10 had different genotype(s) at the two screens, 1 woman tested genotype-negative at the 1999/2002 screen, and 2 women tested genotype-negative at the 2017/2018 screen (Table 1). Among the 14 hrHPV-positive women with known genotype(s) at both screens, 6 had at least one positive intermediate hrHPV test result reported between 6 months and 5 years after enrolment. All of the 12 genotypes detected in those 6 women were also reported at enrolment and only one was also reported after ≥15 years. The 3-year histological follow-up of the 136 hrHPV-positive women at the 2017/2018 screen yielded 11 CIN2 and 10 CIN3 lesions. None of the 11 hrHPV-positive women with CIN2 had a positive hrHPV result at the 1999/2002 screen, while 4 of the 10 with CIN3 cases had a hrHPV-positive result at the 1999/2002 screen (Table 1).

**Table 1:** Cervical hrHPV genotypes at the POBASCAM enrolment screen in 1999/2002 and at the 2017/2018 screen, stratified for genotype concordance and CIN3 in the 3-year follow-up of the 2017/2018 screen.

	Birth year woman	Screen 1999/2002 hrHPV genotype	Screen 2017/2018 hrHPV genotype	
No genotype concordance, no CIN3^a^	1958	16	18, 51, 52	
	1963	39	18, 31	
	1967	16	18, 56	
	1967	51	52	
	1968	16	33, 56	
	1968	16	35	
	1968	31	51	
	1968	45, 56	31	
	1969	16, 45	31	

At least one equal genotype, no CIN3	1968	18, 45, 51, 66	66	
	1968	35	35, 59	
CIN3	1967	16	16, 31	
	1968	45	33	
	1972	16	16	
	1968	hrHPV-pos/genotype-neg	45	
	1968	hrHPV-neg	16	
	1968	hrHPV-neg	33	
	1968	hrHPV-neg	33	
	1968	hrHPV-neg	18, 45	
	1972	hrHPV-neg	16, 45	
	1973	hrHPV-neg	16, 18, 51	

Abbreviations: CIN3, cervical intraepithelial neoplasia grade 3; hrHPV, high-risk human papillomavirus; neg: negative; pos: positive.

a 119 women with a negative hrHPV result in 1999/2002 and a positive hrHPV result in 2017/2018 (and not treated for ≥ CIN2 or hysterectomy in between) and 2 women with a positive hrHPV result in 1999/2002 and a positive (genotype-negative) hrHPV result in 2017/2018 were not included in the table.

Table 2 shows the associations between hrHPV screening results at the two screens ≥15 years apart among women who were not treated for ≥ CIN2 or had a hysterectomy between 1999/2002 and 2017/2018 (N = 2162). Women who were hrHPV-positive in 1999/2002 had a 3.4 times higher odds of being hrHPV-positive in 2017/2018 than women who were hrHPV-negative in 1999/2002 (OR 3.4, 95% CI 1.8–6.1). Despite the association not being statistically significant, women who were HPV16-positive in 1999/2002 had a 5.8 times higher odds of being HPV16-positive in 2017/2018 than women who were HPV16-negative in 1999/2002 (OR 5.8, 95% CI 0.6–25.2). The analyses at the genotype level showed that women who were positive for any hrHPV genotype in 1999/2002 were more likely to have a genotype concordant result (OR 5.1, 95% CI 1.0–11.3) in 2017/2018 and also more likely to have a genotype non-concordant result (OR 3.7, 95% CI 1.6–6.7) in 2017/2018 than those who were genotype-negative in 1999/2002. Positive associations were also observed when stratifying by age. In women <50 years of age, the OR (95% CI) was 3.1 (1.3–6.5) for generic hrHPV, 4.2 (0.1–30.3) for HPV16, 5.7 (0.0–14.7) for pooled genotype-concordant hrHPV, and 3.7 (1.2–7.8) for pooled genotype non-concordant hrHPV. In women aged ≥50 years, ORs were 3.8 (1.4–9.2), 8.5 (0.2–68.3), 3.5 (0.0–13.1), and 3.2 (0.7–7.6), respectively. When including ≥ CIN2 or hysterectomy cases detected before 2017 (N = 2162 + 55 = 2217), ORs were 2.9 (1.7–4.8), 3.3 (0.4–13.8), 3.3 (0.7–7.3), and 3.2 (1.7–5.4), respectively.

**Table 2:** Analysis of screen-detected hrHPV infections in 1999/2002 and in 2017/2018 among women who were not treated for ≥ CIN2 or had a hysterectomy between the two screens (N = 2162).

Screen 1999/2002		Screen 2017/2018		OR (95% CI)^a^
	**Total**	**Positive**	**Negative**	
		hrHPV		
**hrHPV-pos**	98	17	81	**3.4 (1.8–6.1)**
**hrHPV-neg**	2064	119	1945	
		HPV16		
**HPV16-pos**	31	2	29	5.8 (0.6–25.2)
**HPV16-neg**	2131	25	2106	
		hrHPV same genotype		
**hrHPV genotype-pos**	112^b^	4	108	5.1 (1.0–11.3)^c^
**hrHPV genotype-neg**	30115^b^	152	30003	
		hrHPV other genotype		
**hrHPV genotype-pos**	112^b^	17	95	3.7 (1.6–6.7)^c^
**hrHPV-neg**	29008^b^	1366	27642	

Abbreviations: CI, confidence interval; HPV, human papillomavirus; hr, high-risk; neg, negative; pos, positive; OR, odds ratio.

a OR (with 95% CI) were calculated between the hrHPV test results of the 1999/2002 screen (enrolment screen of the POBASCAM study) and of the 2017/2018 screen. Differences in risks were evaluated by means of Fisher's exact test and p < 0.05 was considered statistically significant. Statistically significant OR are depicted in bold.

b Pooled over 14 hrHPV genotypes (listed in 2.2. Laboratory procedures) for 2162 women.

c Mantel-Haenszel OR (with 95% bootstrap CI) is shown for the pooled genotype level analyses (crude OR (95% CI): 7.3 (1.9–19.6) for hrHPV genotype-concordant results and 3.6 (2.0–6.1) for hrHPV genotype non-concordant results).

In the subgroup of women with a positive hrHPV result at the 2017/2018 screen, we found that women who were hrHPV-positive in 1999/2002 had a higher risk of CIN3 than women who were hrHPV-negative in 1999/2002 (OR 5.8, 95% CI 1.0–27.8; p = 0.022). Besides, we found that in the subgroup of women with an HPV16-positive result in 2017/2018, the risk of CIN3 was higher when they also were HPV16-positive in 1999/2002 than when they were HPV16-negative in 1999/2002 (OR ∞, 95% CI 2.9-∞; p = 0.028).

## 4 Discussion

Our study shows that the hrHPV result in screening is associated with detection of hrHPV and CIN3 15–20 years later. The risk of hrHPV detection was elevated for the generic hrHPV result, for infections with the same genotype, and also for infections with genotype(s) different than the one(s) detected earlier.

Our study is unique because it is based on population-based hrHPV screening data with genotyping information at two screens ≥15 years apart and our findings may have important implications for screening management. The association between the hrHPV test result and hrHPV positivity and CIN3 ≥15 years later supports the inclusion of previous hrHPV results in screening recommendations. Based on our analyses, it seems recommendable to screen women every 3–5 years for multiple screening rounds after hrHPV has been detected. Note that in Sweden and the Netherlands, intervals up to 7 and 10 years are currently recommended and our study indicates that these intervals may be reconsidered when a hrHPV infection has been reported in the past. At the genotype level, we were only able to establish a long-term association between an HPV16 infection and CIN3 because of the limited size of the data set. Therefore, the implications for screening are still somewhat unclear for non-HPV16 genotypes, that is, whether long-term intensive screening needs to be maintained for non-HPV16 genotypes. The impact of previous hrHPV results has also been addressed in a US study, where risk stratification based on test results of previous rounds improved the prognostic accuracy for CIN3 and cancer [5,13], supporting the idea that risk-based screening algorithms can be developed to improve both efficiency and efficacy of screening.

Several potential explanations exist for the elevated risk of hrHPV positivity of a different genotype after ≥15 years in women who were hrHPV-positive in POBASCAM. First, hrHPV infections detected at the 2017/2018 screen could have been already present at the start of the POBASCAM study in 1999/2002 but were missed due to technical masking. This explanation is unlikely because the genotypes found between 6 months and 5 years after POBASCAM enrolment were highly concordant with the genotypes found at enrolment, and only one genotype found between 6 months and 5 years was also found in 2017/2018. Second, hrHPV positivity is a marker for sexual behavior, at least at the population level, and therefore the subgroup of hrHPV-positive women may have an elevated risk of acquiring a new hrHPV infection later in life. This conjecture is supported by a Canadian cohort with 10 years of follow-up [4], where new infections were strongly associated with new sexual partners. Third, the chance of observing a positive hrHPV result increases when it takes longer to clear an infection and the population of hrHPV-positives may contain women having difficulties clearing a hrHPV infection. Finally, latency may play a role when hrHPV-positive women also have a higher risk of a latent infection of a different genotype compared to hrHPV-negative women.

In conclusion, we found that a positive hrHPV result in screening gives a long-term increased risk of a hrHPV positive result, also for different genotypes, and a long-term increased risk of CIN3. This supports the idea of risk stratification by including previous hrHPV results in screening programs with primary hrHPV testing.

## Authors’ contributions

FI and JB designed the study. AGS, CJLMM and DAMH provided the data. FI and JB analyzed the data. FI, JAB and JB drafted the manuscript. All authors contributed to the data interpretation and revision of the manuscript giving their contribution to improve it and approved the final version.

## Funding

This project has received funding from the 10.13039/100005622Netherlands Organization for Health Research and Development (ZonMw, project No 50-53125-98-034) and from the 10.13039/501100007601European Union's Horizon 2020 research and innovation program (RISCC project, grant agreement No 847845). The funders had no role in the identification, design, conduct, reporting, and interpretation of the analysis.

## Data availability statement

The data that support the findings of this study are available from the corresponding author upon request.

## Conflict of interest

CJLMM and DAMH declare the following financial interests/personal relationships which may be considered as potential competing interests:

CJLMM is minority shareholder and part-time CEO of Self-screen B.V., a spin-off company of Amsterdam UMC, location VUMC, which develops, manufactures and licenses high-risk HPV assays and methylation marker assays for cervical cancer screening and holds patents on these tests. CJLMM formerly had a small number of shares of Qiagen and MDXHealth. He has received consultancy fees from GlaxoSmithKline, Qiagen, Sanofi Pasteur MSD/Merck, and Asieris/Ismar Healthcare NV, and served occasionally on the scientific advisory boards (expert meetings) of these companies.

DAMH is minority shareholder of Self-screen B.V.

The other authors declare that they have no known competing financial interests or personal relationships that could have appeared to influence the work reported in this paper.
